# Stability of Bacteriocin-Like Inhibitory Substance (BLIS) Produced by* Pediococcus acidilactici* kp10 at Different Extreme Conditions

**DOI:** 10.1155/2018/5973484

**Published:** 2018-09-27

**Authors:** Nurul Lyana Md Sidek, Murni Halim, Joo Shun Tan, Sahar Abbasiliasi, Shuhaimi Mustafa, Arbakariya B. Ariff

**Affiliations:** ^1^Department of Bioprocess Technology, Faculty of Biotechnology and Biomolecular Sciences, Universiti Putra Malaysia, 43400 UPM Serdang, Selangor, Malaysia; ^2^Bioprocessing and Biomanufacturing Research Centre, Faculty of Biotechnology and Biomolecular Sciences, Universiti Putra Malaysia, 43400 UPM Serdang, Selangor, Malaysia; ^3^Bioprocess Technology, School of Industrial Technology, Universiti Sains Malaysia, 11800 Penang, Malaysia; ^4^Department of Microbiology, Faculty of Biotechnology and Biomolecular Sciences, Universiti Putra Malaysia, 43400 UPM Serdang, Selangor, Malaysia; ^5^Halal Products Research Institute, Universiti Putra Malaysia, 43400 UPM Serdang, Selangor, Malaysia

## Abstract

Nowadays, bacteriocin industry has substantially grown replacing the role of chemical preservatives in enhancing shelf-life and safety of food. The progress in bacteriocin study has been supported by the emerging of consumer demand on the applications of natural food preservatives. Since food is a complex ecosystem, the characteristics of bacteriocin determine the effectiveness of their incorporation into the food products. Among four commercial media (M17 broth, MRS broth, tryptic soy broth, and nutrient broth) tested, the highest growth of* Pediococcus acidilactici* kp10 and bacteriocin-like-inhibitory substance (BLIS) production were obtained in the cultivation with M17. BLIS production was found to be a growth associated process where the production was increased concomitantly with the growth of producing strain,* P. acidilactici* kp10. The antimicrobial property of BLIS against three indicator microorganisms (*Listeria monocytogenes*,* Escherichia coli,* and* Staphylococcus aureus*) remained stable upon heating at 100°C but not detectable at 121°C. The BLIS activity was also observed to be stable and active at a wide pH range (pH 2 to pH 7). The BLIS activity remained constant at -20°C and -80°C for 1 month of storage. However, the activity dropped after 3 and 6 months of storage at 4°C, -20°C, and -80°C with more than 80% reduction. The ability of bacteriocin from* P. acidilactici* kp10 to inhibit food-borne pathogens while remaining stable and active at extreme pH and temperature is of potential interest for future applications in food preservatives.

## 1. Introduction

Bacteriocin is defined as a proteinaceous compound produced by microorganisms to inhibit the growth of similar or closely related bacterial strains [1]. To date, a large number of bacteriocins produced from lactic acid bacteria (LAB) from most common genera such as* Lactococcus*,* Streptococcus*,* Pediococcus*,* Leuconostoc*,* Lactobacillus,* and* Carnobacterium* have been identified even though their potential as biopreservatives or antibiofilm agents has not been well established. In fact, there are a few bacteria having the ability to produce more than one bacteriocin and multiply bacteriogenic strains such as* Streptococcus salivarius*,* Streptococcus uberis,* and* Streptococcus mutans* [2].

Biological actions of bacteriocin may be exhibited by either bactericidal or bacteriostatic mode of action [3]. In order to assign which mode of action is expressed by a bacteriocin, several factors need to be considered such as assay systems used, concentrations and purity of the inhibitor, sensitivity of the indicator species, density of cell suspension used, and type of buffer or broth[4]. In general, both mechanisms are influenced by several factors including dose of bacteriocin, degree of purification, presence of other antimicrobial compounds, and physiological state of indicator cells (i.e., growth phase, experimental conditions). Nevertheless, most identified bacteriocins have been reported to use bactericidal action by pore formation as their mechanism towards sensitive organisms. For instance, through membrane permeable assay, salivaricin 9 lantibiotic produced by* Streptococcus salivarius* NU10 was found to penetrate the cytoplasmic membrane and induce pore formation that resulted in cell death [5]. The pore forming mechanism of inhibition was confirmed after samples were visualised using scanning electron microscopy (SEM).

In general, the inhibitory spectrum of bacteriocin can be narrow and confined to closely related species or relatively broad inhibiting a range of food-spoilage and pathogenic bacteria [6]. The need for new bacteriocins with broad antibacterial spectrum for feasible application in foods has substantially increased to replace the role of chemical preservatives in enhancing shelf-life and safety of food [7]. The potential of bacteriocins to inhibit pathogens commonly involved in food-borne illnesses such as* Listeria monocytogenes*,* Staphylococcus aureus*, enteropathogenic* Escherichia coli, Salmonella typhimurium,* and* Shigella dysenteriae* and their toxins is of the major demand in the food industry [8, 9].

Bacteriocin-like-inhibitory substance (BLIS) is often interchangeably used with bacteriocin since BLIS is uncharacterised bacteriocin that apparently shares similar activity. Just like bacteriocin, BLIS activity can also be quantified by various methods such as agar well diffusion assay, spot-on-lawn assay, turbidimetric assay, ELISA, radiometry, conductance measurements, and bioassays based on self-induction skills [10]. However, determination method employing sensitive microorganisms to exhibit the growth inhibition potential of a bacteriocin is the most widely used assay. In general, each bacteriocin/BLIS has its own characteristics that make it useful in its own way. Physicochemical properties of bacteriocin are apparently important in food industry in which it has to be incorporated in a complex environment of food during processing [11].* In vivo *application of BLIS in food model system will provide information on the influence of food properties on BLIS/ bacteriocin production [12]. For example, Scatassa et al. [13] reported on* in vivo* application of BLIS produced by LAB strains isolated from Sicilian dairy environments in several food models including UHT milk, minicheeses, and traditional Sicilian cheeses to investigate their anti-*Listeria *activity. To date, there are a number of BLIS/bacteriocins that have been characterised from various* Pediococcus* species isolated from many sources such as dairy products, meat products, and other fermented products [14]. Generally, BLIS/bacteriocins produced from* Pediococcus *species such* as P. acidilactici, P. pentosaceus,* and* P. damnosus* are mostly small and hydrophobic proteins [15]. Bacteriocins/BLIS produced from pediococci are usually characterised under class IIa group with antilisterial activity properties. In fact, their bactericidal action is often stable to heat treatments, sometimes even at sterilisation temperature [16]. Their activities can retain in cold temperature of -80°C and are also sensitive to most proteases.

Biopreservation method using bacteriocin has been extensively chosen since it maintains the organoleptic and nutritional properties even with a little use of chemical preservatives and lower heat intensity [17]. However, the actions of bacteriocin in inhibiting growth of pathogens are not easy to predict and describe [18]. Suitable media composition and culture conditions must be used in LAB fermentation not only to enhance the growth [19–21], but also to boost the secretion of bacteriocin [22]. In medium formulation, it is crucial to investigate the roles of all components to develop processes that are inexpensive, rapid, environmental friendly, highly yielding, and amenable to a large scale production. In addition, production of BLIS/bacteriocin may rely on the aeration or oxygen supply. Strict aerobes and strict anaerobes can only survive in either presence or absence of oxygen while facultative anaerobic organisms are able to adapt to changes in the environmental oxygen concentration and accordingly shifting their cellular metabolism [23]. Production of bacteriocin by* B. subtilis *was dependent on aeration conditions and the maximum production was recorded under reduced aeration compared to low aeration [24].

The key obstacle of bacteriocin applications in the food industry is the food ecosystem [25]. Different types of food have different complex ecosystems. In order for the bacteriocin to be incorporated into food system, the physicochemical properties need to be firstly studied especially in terms of stability to temperature, pH, storage duration, and other factors that may influence its activity. The present study was hence carried out to investigate the effects of several commercial basal media on the growth of* Pediococcus acidilactici* kp10 and bacteriocin production to enhance the performance of bacteriocin fermentation in terms of final activity, yield, and overall productivity. The stability of BLIS produced by* P. acidilactici* kp10 was also evaluated under different extreme temperatures, pH, and storage periods with several food-borne pathogens as indicator microorganisms.

## 2. Materials and Methods

### 2.1. Bacterial Strains

The LAB, which is* P. acidilactici* kp10, previously isolated from traditionally prepared milk product (dried curd) [26] was used throughout this study.* L. monocytogenes *ATCC 15313*, S. aureus* ATCC 33591, and* E. coli* ATCC 35218 were used as indicator microorganisms for the quantification of bacteriocin-like inhibitory substance (BLIS) produced by* P. acidilactici* kp10. All strains were maintained in glycerol (20% v/v) at -80°C as stock culture. Purity of cultures was maintained by streaking method on agar medium. Prior to use, the bacteria were subcultured twice in an appropriate media at 37°C for 24 h without agitation.

### 2.2. Inoculum Preparation

The primary culture was prepared by growing a single bacterial colony from an agar plate and grown in 50 mL tube containing 10 mL of M17 broth (MERCK, Germany). It was then incubated at 37°C without agitation for 24 h. For inoculum preparation, 1% (v/v) of the primary culture was inoculated into a 50 mL tube containing 10 mL of M17 medium, incubated in orbital shaker at 37°C and agitated at 100 rpm for 24 h. This inoculum that contains 10^8^ CFU/mL as determined using plate count method, corresponding to optical density of 0.03 read at 600 nm, was used in all experiments.

### 2.3. Shake Flask Fermentation

All shake flask fermentation was initiated by inoculating 1% (v/v) of inoculum, prepared as earlier described in Section 2.2. The flasks were incubated in an orbital shaker at 28.5°C and agitated at 120 rpm, 28 h condition, which was the optimal condition for growth of* P. acidilactici* kp10 as previously screened and optimised using Response Surface Methodology (RSM) and Artificial Neural Network (ANN) [27].

### 2.4. Effects of Commercial Media on Growth of P. acidilactici kp10 and BLIS Production

Four types of commercial basal medium, (i) tryptic soy broth (TSB), (ii) nutrient broth (NB), (iii) MRS broth, and (iv) M17 broth (MERCK, Germany), were preliminary tested for the growth of* P. acidilactici* kp10 and BLIS production as fermentation conditions described in Section 2.3. NB consists of peptones from meat (15g/L), yeast extract (3g/L), sodium chloride (6g/L), and D-(+)-glucose (1g/L). TSB comprised peptone from casein (17g/L), peptone from soymeal (3g/L), D-(+)-glucose (2.5g/L), sodium chloride (5g/L), and di-potassium hydrogen phosphate (2.5g/L). MRS consists of peptone from casein (10g/L), meat extract (8g/L), yeast extract (4g/L), D-(+)-glucose (20g/L), di-potassium hydrogen phosphate (2g/L), Tween® 80 (1g/L), di-ammonium hydrogen phosphate (2g/L), sodium acetate (5g/L), magnesium sulphate (0.2g/L), and manganese sulphate (0.04g/L). On the other hand, M17 consists of peptone from soymeal (5g/L), peptone from meat (2.5g/L), peptone from casein (2.5g/L), yeast extract (2.5g/L), meat extract (5g/L), D-(+)-lactose (5g/L), ascorbic acid (0.5g/L), Na-*β*-Glycerophosphate (19g/L), and magnesium sulphate (0.25g/L). From this study, it was found that M17 was the preferred medium for growing* P. acidilactici* kp10 and BLIS production. Thus, M17 medium was used in all subsequent experiments.

### 2.5. Effects of Different Concentrations of M17

The effects of different concentrations of M17 medium on the growth of* P. acidilactici* kp10 and BLIS production were also investigated. All conditions were either prepared accordingly by diluting the media to 75%, 50%, and 25% or concentrated by adding 25% and 50% extra M17 to observe its influence on growth and BLIS production.  Table 1 shows the percentage of M17 concentrations used in the experiment.

### 2.6. Preparation of Crude BLIS

The cultures at the end of fermentation were centrifuged at 13,000 x g for 10 minutes at 4°C for separation of cells, according to the method described by Udhayashree et al. [28]. The cell-free supernatant termed as crude BLIS was used for the determination of BLIS activity.

### 2.7. Analytical Procedures

#### 2.7.1. Cell Growth

Cell growth was determined by measuring the optical density (OD) at a wavelength of 600 nm using spectrophotometer (GENESYS 20, Thermo Scientific, UK) with 0.5 % (w/v) NaCl solution as a blank. A known volume of sample was centrifuged using microcentrifuge (Thermo Scientific Heraeus Pico 21, USA) at 18,894g rpm for 10 min. The same volume of 0.5 % (w/v) NaCl as that of supernatant volume removed was added to the cell pellet and vortexed. The cells were then washed with 0.5 % (w/v) NaCl prior to transferring them to a plastic cuvette. Cell density was measured by absorbance at 600 nm using GENESYS ™ 20 spectrophotometer (Thermo Fisher Scientific, USA).

Cell growth was determined by filtration and oven dried method and expressed as dry cell weight (DCW). For dry cell weight, 5 mL of supernatant was filtered by vacuum application through preweighed 0.45 *μ*m pore size filter membranes (Whatman, UK). Once filtered, the membranes were dried in an oven at 80°C until constant weight was achieved at least for 48 h. Equation (1) [29] was used to obtain the dry weight of the cell (g/L).

(1) $$\begin{array}{c} Dry\;\;cell\;\;weight\left(\;g/L\;\right)=\frac{Dry\;\;weight\;\;\text{of}\;\;\text{filter}\;\;paper+cell\left(g\right)-Dry\;\;weight\;\;\text{of}\;\;\text{filter}\;\;paper\left(g\right)}{0.005L} \end{array}$$

#### 2.7.2. Determination of BLIS Activity

**Figure 1:**
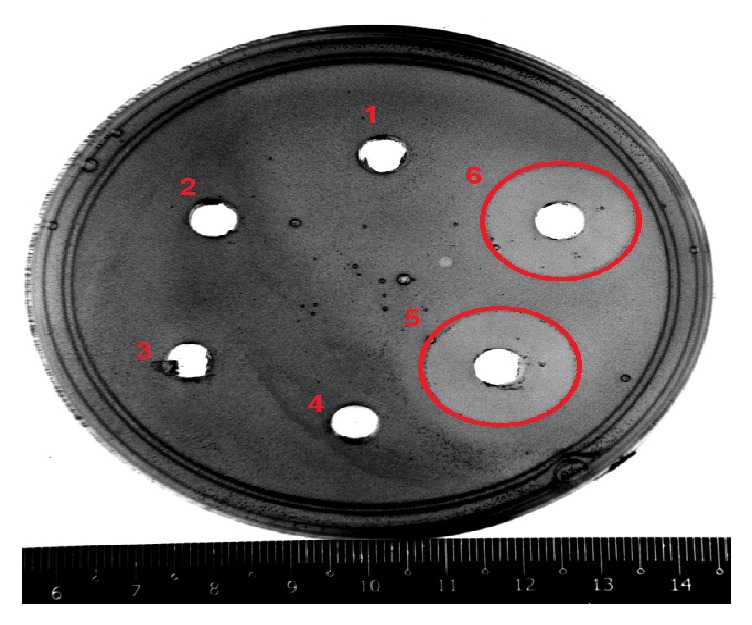
Antimicrobial activity of crude free cell supernatant from* P. acidilactici* kp10 against* L. monocytogenes* ATCC 15313. Inhibition zone observed on the agar plate of agar well diffusion method is marked by red circle. 1: media; 2: media; 3: distilled water; 4: distilled water; 5: crude free cell supernatant; 6: crude free cell supernatant.

In this study, BLIS activity was measured by agar well diffusion assay method (AWDA) [30]. The crude BLIS was tested upon* L. monocytogenes* ATCC 15313 as indicator organism. Aliquots of 100 *μ*L crude BLIS were pipetted into 6 mm diameter well of cooled soft TSB seeded with* L. monocytogenes* ATCC 15313. The plates were incubated at 37°C for 24 h. The diameters of zone of inhibition were then measured (Figure 1). The BLIS activity was defined as AU that is equivalent to the unit area of inhibition zone per unit volume (expressed as mm^2^/mL) as

(2) $$\begin{array}{c} BLIS\;\;activity=\left(\;\frac{{mm}^{2}}{mL}\;\right)=\frac{Lz-Ls}{V} \end{array}$$

where

 
L_z_ = clear zone area (mm^2^)
 
L_s_ = well area (mm^2^)
  V = volume of sample (mL)

### 2.8. Determination of BLIS Stability at Different Conditions

In this set of experiment, the culture of 24 h BLIS fermentation by* P. acidilactici* kp10, carried out in shake flask incubated in an orbital shaker at 28.5°C and agitated at 120 rpm, was used for the determination of BLIS stability at different conditions. The culture was centrifuged to separate the cell for preparing crude BLIS according to the method as described earlier.

#### 2.8.1. Effects of Temperature on BLIS Stability

To investigate the effects of temperature on BLIS stability, the crude extract of BLIS in Eppendorf tubes (15 mL) was incubated at different temperatures: 4°C, - 20°C, and - 80°C for 24 h and 100°C and 121°C for 15 minutes (to mimic the most commonly used conditions for autoclave method to sterilise equipment and supplies). At the end of each incubation, BLIS activity was determined according to the method previously described, upon three indicator microorganisms (*L. monocytogenes* ATCC 15313,* E. coli* ATCC 35218, and* S. aureus* ATCC 33591).

#### 2.8.2. Effects of pH on BLIS Stability

The activity of BLIS at pH of 2 to 9 was monitored only for* L. monocytogenes* ATCC 15313 as an indicator microorganism. The pH of the crude BLIS (pH 6.5) was adjusted to pH ranging from 2 to 9 using various buffer systems by adding either 1 M NaOH or 1 M HCl according to method as described by Miao et al. [31]. All of the samples were stored in 15 mL Falcon tubes at room temperature. The crude BLIS after pH adjustment was incubated at 37°C for 24 h and BLIS activity was determined immediately after sampling.

#### 2.8.3. Effects of Storage Periods on BLIS Stability

The activity of BLIS during storage at 4°C, -20°C, and -80°C and pH from 2 to 9 for 1, 3, and 6 months was monitored using only* L. monocytogenes* ATCC 15313 as an indicator microorganism.

### 2.9. Statistical Analysis

A one-way analysis of variance (ANOVA) with Tukey's test was used to test the significance differences among treatment means.

## 3. Results

### 3.1. Effects of Commercial Media on Growth of P. acidilactici kp10 and BLIS Production

**Figure 2:**
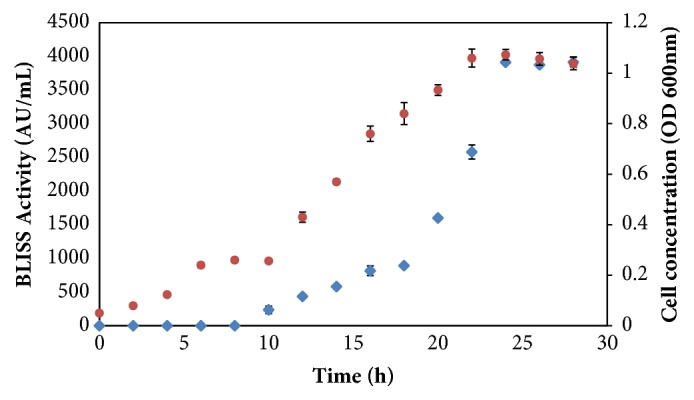
The profiles of BLIS activity (◆) and cell concentration (●) of* P. acidilactici* kp10 in M17 media. The error bars represent the standard deviations about the mean (n=3).

Table 2 displays the effects of different basal media namely M17, de Man, Rohosa, Sharpe (MRS), tryptic soy broth (TSB), and nutrient broth (NB) on the growth of* P. acidilactici* kp10 and BLIS activity. In all cases, the final pH was found to be slightly increased from the initial culture pH. For all fermentation, maximum BLIS activity was achieved after 24 h. The highest growth of* P. acidilactici *kp10 (OD = 1.025) and BLIS activity (3986.91 AU/mL) was obtained in M17 medium. Meanwhile, BLIS activity after 24 h fermentation was observed to be 19, 10, and 29 times higher than that obtained in MRS (215.38 AU/mL), TSB (386.09 AU/mL), and NB (134.73 AU/mL), respectively. In general, BLIS production was high with increased concentration of* P. acidilactici* kp10 cell in the culture. However, the ability of the* P. acidilactici* kp10 cell in producing BLIS was seen varied with the use of different basal media. As shown by the BLIS yield based on cell present in the culture, the highest yield (10.49x 10^2^AU/g cell) was obtained in fermentation with M17 medium, followed by TSB (6.43 x 10^2^AU/g cell), NB (2.69 x 10^2^ AU/g cell), and MRS (1.79 x 10^2^ AU/g cell) media. The highest productivity (43.71 AU/g cell/h) was obtained in fermentation using M17 medium, followed by TSB (26.79 AU/g cell/h), MRS (7.46AU/g cell/h), and NB (11.21 AU/g cell/h) media. Based on the profiles of BLIS activity and cell concentration of* P. acidilactici* kp10 in M17 media as shown in Figure 2, BLIS activity was only detected after 10 h of fermentation and stopped after the growth reached a stationary growth phase. This suggests that BLIS production by* P. acidilactici* kp10 is a growth associated process.

### 3.2. Effects of Different M17 Concentrations on Growth of P. acidilactici kp10 and BLIS Production

Based on the highest growth and BLIS activity attained in Section 3.1, the effects of different M17 concentrations (25%-150%) on the growth of* P. acidilactici* kp10 and BLIS production were further investigated as summarized in Table 3. The growth of* P. acidilactici* kp10, as measured by cell number and dry cell weight was slightly similar to fermentation with M17 concentration ranging from 50 to 100%. Likewise, BLIS activities obtained in this fermentation were also comparable to one another at 2855.33 AU/mL. It is interesting to note that a substantial reduction in BLIS activity (1189.67 AU/mL) was recorded in the fermentation with 25% of M17, which was 140% lower than that obtained in the fermentation with 50 to 100% M17. Meanwhile, a slight reduction in growth and BLIS production was observed in the fermentation with high M17 concentrations (125 to 150%), suggesting that inhibition of growth of* P. acidilactici* kp10 and BLIS production may occur at high carbon and nitrogen concentrations.

### 3.3. BLIS Stability at Different Temperatures

The effects of incubation temperature on BLIS stability are tabulated in Table 4. No BLIS activity was detected after the exposure to high temperature (121°C) for 15 minutes as determined using all three types of indicator microorganism (*L. monocytogenes* ATCC 15313,* E. coli* ATCC 35218, and* S. aureus* ATCC 33591). The BLIS activity measured using* L. monocytogenes* ATCC 15313 after exposure to temperature of 4°C for 24 h remained the same as the activity of crude BLIS (2327.75 AU/mL). Similar result was observed on BLIS exposed to high temperature (100°C) for 15 minutes, suggesting that BLIS of* P. acidilactici* kp10 is heat stable. For BLIS exposed to a very low temperatures (-20°C and -80°C) for 24 h, the activity was seen reduced by half of the initial activity of crude BLIS.

The antimicrobial activity of BLIS towards* E. coli* ATCC 35218 (189.21 AU/mL) was lower than that towards* L. monocytogenes* for crude BLIS stored at 4°C for 24 h. Similar to activity measured using* L. monocytogenes*, the BLIS activity after the exposure to 100°C for 15 minutes was significantly higher compared to that measured for BLIS stored at -20°C and -80°C for 20 min.

In the meantime,* S. aureus *exhibited the lowest antimicrobial activity (52.39 AU/mL) for crude BLIS stored at 4°C for 24 h compared to other indicator microorganisms. Reduction in BLIS activity (around 35 AU/mL) for about 40% was also recorded for crude BLIS stored at -20°C and -80°C for 24 h. BLIS activity was not detected when BLIS was exposed to high temperatures of 100°C and 121°C for 15 minutes. Based on the large discrepancy observed in the antimicrobial activity of BLIS towards* L. monocytogenes *and the other two indicator microorganisms,* E. coli *and* S. aureus,* therefore, the subsequent experiments were only conducted for* L. monocytogenes*.

### 3.4. BLIS Stability at Different pH

**Figure 3:**
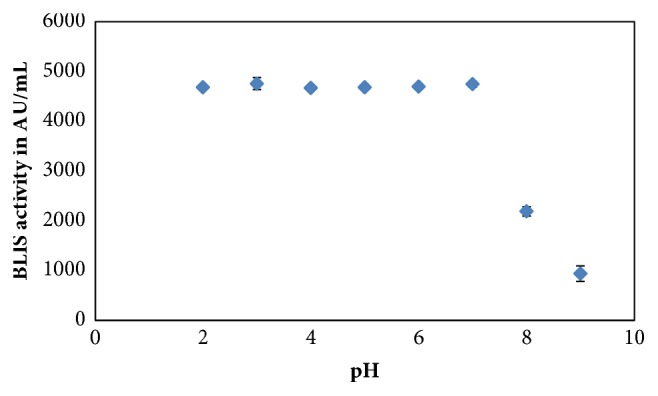
The effect of exposure to various pH on BLIS stability towards* L. monocytogenes* ATCC 15313. The error bars represent the standard deviations about the mean (n=3).

Figure 3 shows the effects of different pH on the stability of BLIS upon the use of* L. monocytogenes* ATCC 15313 as an indicator microorganism. The BLIS produced by* P. acidilactici* kp10 was stable when exposed to a wide range of pH (pH 2 to 7) during a period of 24 hours where the activity remained similar to that of crude BLIS at pH 6.5 (4689.2 AU/mL). Substantial reduction in BLIS activity was observed when exposed to high pH (2132.62 AU/mL for pH 8 (54.5% reduction in activity) and 814.07 AU/mL for pH 9 (82.6% reduction in activity)), indicating that the BLIS produced by* P. acidilactici* kp10 is not stable at alkaline pH.

### 3.5. BLIS Stability during Long Term Storage

The stability of BLIS produced by* P. acidilactici* kp10, using* L. monocytogenes* ATCC 15313 as an indicator microorganism during long term storage at various temperatures is summarized in Table 5. The activity of BLIS (2132.62 AU/mL) stored at 4°C remained constant after 1 month. However, the activity (370.22 AU/mL) was tremendously diminished after 6 months, which corresponded to 82.64% of activity reduction. During storage at -20°C and -80°C, the BLIS activity decreased by half after 1 month and further declined to 276.51 AU/mL after 6 months, which corresponded to more than 87% reduction in activity.

## 4. Discussion

Results from this study demonstrated that medium formulation and composition greatly influence the growth of* P. acidilactici* kp10 and its ability to secrete BLIS. The influence may come from the qualitative nature of the nutrient sources of carbon and nitrogen [22]. In most cases, a number of studies have noted that good cell growth has a direct correlation with BLIS production [32]. However, optimal cell growth does not always result in high bacteriocin production. Generally, a low growth rate or unfavourable conditions may also stimulate bacteriocin production [33]. The maximum BLISS activity in TSB (absent of Tween 80) was higher than in MRS (presence of Tween 80) despite the twofold lower maximum cell growth observed in TSB. Some LAB strains require surfactant such as Tween 80 or Tween 20 as a fatty acid supplement to support their growth [34]. As reported by Ca'lix-Lara et al. [35] several LAB grew with an equal efficiency in MRS and TSB supplemented with Tween 80. The presence of surfactant may enhance the sensitivity of the indicator strain and form micelles with proteinaceous compounds to stabilise bacteriocin [36]. The addition of surfactants such as hexadecyltrimethylammonium bromide, ethylenediaminetetraacetic (EDTA), and sodium dodecyl sulphate (SDS) to crude bacteriocin from* Lactobacillus acidophilus* NCIM5426 has increased the antibacterial activity against food-borne (*L. monocytogenes* and* S. aureus*) and human pathogens (*E. coli* and* S. typhi*) [37]. Nonetheless, the antibacterial activity was decreased in the presence of metal ions such as mercuric chloride, zinc sulphate, and calcium chloride. Hence, supplementation of surfactant in basal media such as TSB and NB is expected to significantly increase the yield of LAB cells and may subsequently increase the BLISS activity.

MRS is often found to be the most prominent medium for LAB growth and bacteriocin production [38]. However, in the present study, high growth (OD at 600nm; 1.025) and BLIS activity (3986.91 AU/mL) were recorded when* P. acidilactici* kp10 was grown in M17 broth compared to MRS, NB, and TSB. These observations suggest that specific nutrients are required for the production of BLIS which may be related to five types of nitrogen sources present in M17 broth, namely, peptone from soy, peptone from casein, peptone from meat, yeast extract, and meat extract. Meanwhile, only two or three nitrogen sources were present for MRS (peptone from casein, meat extract, and yeast extract), TSB (peptone from casein and peptone from soy), and NB (peptone from meat and yeast extract). Furthermore, it was found that* P. acidilactici* kp10 preferred lactose (carbon source in M17) instead of glucose (carbon source in NB, TSB, and MRS) as the carbon source. Likewise, bacteriocin avicin A from* E. avium* against* Lactobacillus sakei *LMGT 2312 displayed a higher activity when the strain was grown with lactose than glucose [39]. The stability of bacteriocin avicin A was also improved by the application of nanotechnology using layered double hydroxide. Lactose was also the preferred carbon source for BLIS production by four LAB strains (KTW2G, KTWsL, TS9S17, and TS9S19) isolated from mangrove forest in southern Thailand [40]. Further, the antibacterial peptide production by* Bacillus licheniformis* AnBa9 was induced by the presence of lactose but gradually decreased with increasing lactose concentration [41]. Several studies have been conducted on optimising the growth medium with respect to bacteriocin production. However, bacteriocins are usually produced in complex media [32, 42, 43]; some of them have been produced at high concentrations in relatively simple broths [44]. Only a few bacteriocins have been studied in defined medium [45, 46]. Nonetheless, since LAB are fastidious with respect to nutrient requirements, a rich medium with yeast extract and protein hydrolysates is often required for good growth and bacteriocin production [38].

Results from this study demonstrated that BLIS activity (115.21AU/mL) in batch fermentation was only detected at the beginning of exponential growth phase (10 h) and reached its maximum activity (3986.91 AU/mL) during the initial stationary phase. Similar observations have been reported by other studies. For instance, in batch fermentation,* Enterococcus faecium* EF55 started to secrete bacteriocin at the early logarithmic phase (200 AU/mL) and achieved a maximum activity (12800 AU/mL) at early stationary phase [47]. Most studies claimed that BLIS production is always correlated with the growth of producers supporting that the volumetric production depends on the total biomass formation [48]. Similar observation was reported for the production of BLISS from* P. acidilactici* strain where it displayed primary metabolite kinetics with the rate of production parallel to the growth rate [49].

When considering a new bacteriocin/BLIS, one of the vital criteria to be evaluated is the ability to withstand thermal treatment. This characteristic determines its usefulness in food system especially in food manufacturing process [7]. If the bacteriocin/BLIS is heat labile, it can resort to adverse effects on the bioactive capability of a bacteriocin, potentially making it less effective. The activity of BLIS produced from* P. acidilactici* kp10 was fairly stable when exposed at 100°C for the activities measured upon* L. monocytogenes* ATCC 15313 and* E. coli* ATCC 35218. The BLIS activity was totally lost when exposed to the temperature of 121°C upon all tested indicator microorganisms. This attribute should be taken into consideration during BLIS application to avoid denaturation effect upon processing steps such as autoclave sterilisation. These characteristics are similar to bacteriocin-like peptides produced from* B. licheniformis* ZJU12 that completely lost its activity at 121°C for 15 minute [50]. The results also portrayed the characteristics of BLIS produced from pediococci for its antilisterial activity since the highest activity was recorded upon* L. monocytogenes*. Meanwhile, the inhibitory activity of BLIS (MBF10-2) from* Streptococcus macedonicus* MBF10-2 was found stable when heated up to 60°C for 30 min, but lost when the BLIS was heated up to 80°C [51]. The retained activity of BLIS produced from* P. acidilactici* kp10 at 4°C for all indicator microorganisms indicates that the BLIS can maintain its activity despite relatively low temperature. This condition is beneficial since most food products are kept at 4°C for storage in food industry. All indicator microorganisms showed reduction to half of activity at -20°C and -80°C. The result from this study was comparable to that of the previous study conducted for characterising pediocin SA-1 from* P. acidilactici* NRRLB5627 [52].

The BLIS activity of* P. acidilactici* kp10 remained unchanged when exposed to pH ranging from pH 2 to pH 7 during incubation period of 24 hours but impaired when exposed to pH 8 and 9. Previously, BLIS activity of* P. acidilactici* kp10 was reported to be stable at pH 2 to 9 after 1-hour incubation [26]. These results showed that the stability of BLIS produced by* P. acidilactici* kp10 at the same range of pH has declined over a period of time. Nonetheless, it can be deduced that the BLIS is more stable at acidic pH but becomes inactive at alkaline pH. This result is in accordance with the activity of BLIS from* Streptococcus macedonicus* MBF10-2 against indicator strain* Leuc. mesenteroides* that was found optimum at pH between 6 and 7 and unaffected by acidic condition but appeared to be abolished at pH above 8 [51]. The impaired activity recorded might be due to proteolytic degradation or protein aggregation [53]. Besides, several pediocins produced by* Pediococcus *spp. were stable at pH ranging from 2 to 10 but reduced in activity at pH above 12. This phenomenon was observed for bacteriocin HA-6111-2 and HA-5692-3 [54]. Meanwhile, the activity of bacteriocin F1 produced by* Lactobacillus paracasei* subsp. Tolerans FX-6 was recorded against* E. coli *as indicator microorganism at pH 3, 6, and 9 with the activity that remained stable as in fresh bacteriocin [31]. However, its activity was totally lost at pH 12. Negative effect of bactericidal activity might occur due to the presence of NaOH and HCl residues during pH adjustment. Besides, some studies reported that the optimum activity of bacteriocin is often closely followed by the optimum pH of the indicator organisms. For instance, the highest antibacterial activity of crude bacteriocin from* Lactobacillus* species isolated from fermented maze (Ogi) was recorded at pH 5 and pH 6, which were the optimum pH for* S. typhimurium* and* S. dysenteriae* [9]. Another desirable criterion for bacteriocins as biopreservative agents is stable at various storage conditions [55]. During one-month storage period, crude BLIS of* P. acidilactici* Kp10 was only stable at 4°C but surprisingly lost 25% of its activity at -20°C and -80°C. This observation contradicted with several other studies reporting that the most appropriate preservation temperature for bacteriocins during long term storage was at freezing temperatures (-20°C or lower) [56, 57]. Moreover, pediocin SM-1 from P. pentosaceus Mees 1934 purified by tricine-SDS-polyacrylamide gel electrophoresis showed the stability in its activity even after 1 year of storage at -20°C and -80°C, suggesting the potential of pediocin SM-1 to be used for prolonged storage [52]. Nevertheless, the stability of pediocin SM-1 in crude form was not evaluated. Thus, it is interesting to conduct a comparative study on the stability between crude, partially purified, and purified forms of bacteriocin from* P. acidilactici* kp10 to further characterise the compound prior to commercial applications. Purified or partially purified bacteriocins of LAB origins can be obtained from various techniques of purification based on their affinity to organic solvents and solubility variations in concentrated salt solution and pH [58–62].

## 5. Conclusions

M17 has served as the preferred medium for growth of* P. acidilactici* kp10 and bacteriocin production. The BLIS production was inhibited in fermentation with high initial concentration of carbon and nitrogen sources. Crude BLIS of* P. acidilactici* kp10 can be considered as heat stable, where the antimicrobial activity against* L. monocytogenes*,* E. coli,* and* S. aureus* remained stable after exposure to high temperature (100°C). The crude BLIS was also stable and active at a wide pH range (pH 2 to pH 7) and after storage at 4°C for 1 month.

## Figures and Tables

**Table 1:** Dilution and concentration of M17 medium. Amount of nitrogen (soymeal (5g/L), peptone from meat (2.5g/L), peptone from casein (2.5g/L), yeast extract (2.5g/L), and meat extract (5g/L)) in M17:17.5 g/L. Amount of carbon in M17:D-(+)-lactose:5.0 g/L.

**Concentration of M17 **	**Amount of Nitrogen (g/L)**	**Amount of Carbon (g/L)**
(75% diluted: 9.3 g/L)	4.38	1.25

(50% diluted: 18.6 g/L)	8.75	2.50

(25% diluted: 27.9g/L)	13.13	3.75

M17 (control) (equivalent to 100%)	17.50	5.00

Addition of 25% extra m17 (37+9.3 g/L) of media (equivalent to 125%)	21.88	6.25

Addition of 50% extra M17 (37 + 18.6 g/L) of media (equivalent to 150%)	26.25	7.50

**Table 2:** Effect of different basal media on growth of *P. acidilactici* kp10 and BLIS activity.

**Performance**	**Basal Medium**			
	**M17**	**deMann Rogosa Sharpe (MRS)**	**Tryptic Soy** **Broth (TSB)**	**Nutrient Broth (NB)**
**Maximum Cell Growth (** **O**.**D**_600**nm**_**)**	1.025 (±0.02)^a^	0.322 (±0.03)^b^	0.167 (±0.04)^c^	0.135 (±0.04)^c^

**Dry Cell Weight (g/L)**	3.8 (±0.01)^a^	1.2 (±0.01)^b^	0.6 (±0.01)^c^	0.5 (±0.02)^c^

**Maximum BLIS Activity (AU/mL)**	3986.91 (±1600.30)^a^	215.38 (±61.13)^b^	386.09 (±154.97)^b^	134.73 (±27.61)^b^

**Initial pH**	7.12	5.25	6.89	6.51

**Final pH**	7.17	6.04	7.55	6.62

**Yield (AU/g cell)**	10.49 x 10^2^(±0.40)^a^	1.79 x 10^2^(±0.14)^d^	6.43 x 10^2^(±0.11)^b^	2.69 x 10^2^ (±0.78)^c^

**Productivity ** **(AU/g cell/h)**	43.71 (±0.57)^a^	7.46 (±0.63)^d^	26.79 (±0.71)^b^	11.21 (±0.55)^c^

^a,b,c,d^Means values in the same row expressed with different superscript letters are significantly different at P< 0.05.

**Table 3:** Effect of different concentrations of M17 medium on growth of *P. acidilactici* kp10 and BLIS production. The results were recorded after 24 h of fermentation.

**Concentration of M17 (**%**)**	**Cell Growth (** **O** **D**_600**nm**_ **)**	**Dry Cell Weight (g/L)**	**pH**	**BLIS Activity (AU/mL)**	**Yield (AU/g cell)**	**Productivity (AU/g cell/h)**
25	0.78 (±0.07)^d^	2.9 (±0.03)^c^	6.88 (±0.01)^a^	1189.67 (±163.08)^d^	4.1 x 10^2^ (±0.18)^d^	17.08 (±0.67)^d^

50	1.17 (±0.02)^a^	4.4 (±0.01)^a^	6.70 (±0.08)^a^	2855.33 (±185.45)^a^	6.5 x 10^2^ (±0.13)^b^	27.08 (±0.07)^b^

75	1.17 (±0.05)^a^	4.3 (±0.02)^a^	6.43 (±0.07)^b^	2855.33 (±175.63)^a^	6.6 x 10^2^ (±0.07)^b^	27.50 (±0.09)^b^

100	1.19 (±0.00)^a^	4.4 (±0.00)^a^	6.45 (±0.06)^b^	2855.33 (±181.71)^a^	6.5 x 10^2^ (±0.17)^b^	27.08 (±0.05)^b^

125	1.04 (±0.02)^b^	3.6 (±0.02)^b^	6.53 (±0.01)^b^	1843.07 (±36.71)^c^	5.1 x 10^2^ (±0.15)^c^	21.25 (±0.14)^c^

150	0.95 (±0.00)^c^	3.5 (±0.00)^b^	6.50 (±0.02)^b^	2467.66 (±94.71)^b^	7.1 x 10^2^ (±0.21)^a^	29.58 (±0.74)^a^

The results presented are the average of triplicate experiments and are expressed as mean ± standard deviation. ^a,b,c,d^Means values in the same column expressed with different superscript letters are significantly different at P< 0.05.

**Table 4:** The activity of crude BLIS after exposure to different temperatures with different indicator microorganisms (*L. monocytogenes* ATCC 15313, *E. coli* ATCC 35218, and *S. aureus* ATCC 33591).

**Temperature**	**BLIS Activity in AU/mL**		
	***L. monocytogenes*** **ATCC 15313**	***E. coli *ATCC** **35218**	***S. aureus*** **ATCC 33591**
Control (crude BLIS)	4689.42 (±160.00)^a^	189.21 (±14.00)^a^	52.39 (±8.00)^a^

4°C (24 h)	4689.42 (±153.00)^a^	189.21 (±12.00)^a^	52.39 (±5.50)^a^

- 20°C (24 h)	2327.75 (±75.00)^b^	64.27 (±5.00)^c^	35.85 (±3.00)^b^

- 80°C (24 h)	2327.75 (±96.00)^b^	64.27 (±3.00)^c^	35.85 (±4.50)^b^

100°C (15 mins)	4689.42 (±150.00)^a^	154.25 (±11.20)^b^	ND

121°C (15 mins)	ND	ND	ND

ND=nondetectable.

^a,b,c^Means values in the same row expressed with different superscript letters are significantly different at P< 0.05.

**Table 5:** Stability of BLIS during long term storage at different temperatures. The activity was measured using *L. monocytogenes* ATCC 15313 as an indicator microorganism.

**Temperature**	**Storage Time (Month)**	**BLIS Activity in (AU/mL)**	%** Reduction in Activity**
4°C	1	2132.62 (±1.15)^a^	0
	3	1189.67 (±0.00)^c^	44.22
	6	370.22 (±1.15)^d^	82.64

-20°C	1	1592.83 (±2.00)_b_	25.31
	3	1189.67 (±1.53)^c^	44.22
	6	276.51 (±1.54)^e^	87.03

-80°C	1	1592.83 (±2.00)^b^	25.35
	3	1189.67 (±1.53)^c^	44.22
	6	276.51 (±1.54)^e^	87.03

^a,b,c,d,e^Means values in the same column expressed with different superscript letters are significantly different at P< 0.05.

## Data Availability

The data used to support the findings of this study are available from the corresponding author upon request.

## References

[1] Mokoena M. P. (2017). Lactic acid bacteria and their bacteriocins: classification, biosynthesis and apllications against uropathogens: A mini-review. *Molecules*.

[2] Jones R. J., Zealand N., Wescombe P. A. (2010). *Protective Cultures, Antimicrobial Metabolites and Bacteriophages for Food and Beverage Biopreservation*.

[3] Prudêncio C. V., dos Santos M. T., Vanetti M. C. D. (2015). Strategies for the use of bacteriocins in Gram-negative bacteria: relevance in food microbiology. *Journal of Food Science and Technology*.

[4] Jeevaratnam K., Jamuna M., Bawa A. S. (2005). Biological preservation of foods. *Journal of Biotechnology*.

[5] Barbour A., Philip K., Muniandy S. (2013). Enhanced Production, Purification, Characterization and Mechanism of Action of Salivaricin 9 Lantibiotic Produced by Streptococcus salivarius NU10. *PLoS ONE*.

[6] Wu J., Wu H., Li P., Lu C. (2016). HIV/STIs risks between migrant MSM and local MSM: a cross-sectional comparison study in China. *PeerJ Preprints*.

[7] Silva C. C., Silva S. P., Ribeiro S. C. (2018). Application of Bacteriocins and Protective Cultures in Dairy Food Preservation. *Frontiers in Microbiology*.

[8] Arqués J. L., Rodríguez E., Langa S., Landete J. M., Medina M. (2015). Antimicrobial activity of lactic acid bacteria in dairy products and gut: Effect on pathogens. *BioMed Research International*.

[9] Onwuakor C., Nwaugo V., Nnadi C., Emetole J. (2014). Supernatant (bacteriocin) production from four Lactobacillus species isolated from locally fermented maize (ogi). *American Journal of Microbiological Research*.

[10] Macaluso G., Fiorenza G., Gaglio R., Mancuso I., Scatassa M. L. (2016). In vitro evaluation of bacteriocinlike inhibitory substances produced by lactic acid bacteria isolated during traditional sicilian cheese making. *Italian Journal of Food Safety*.

[11] Papagianni M., Anastasiadou S. (2009). Pediocins: The bacteriocins of Pediococci. Sources, production, properties and applications. *Microbial Cell Factories*.

[12] Settanni L., Guarcello R., Gaglio R. (2014). Production, stability, gene sequencing and in situ anti-listeria activity of mundticin KS expressed by three enterococcus mundtii strains. *Food Control*.

[13] Scatassa M. L., Gaglio R., Cardamone C. (2017). Anti-listeria activity of lactic acid bacteria in two traditional sicilian cheeses. *Italian Journal of Food Safety*.

[14] Abdelhadi A. A., Elarabi N. I., Salim R. G., Sharaf A. N., Abosereh N. A. (2016). Identification, characterization and genetic improvement of bacteriocin producing lactic acid bacteria. *Biotechnology*.

[15] Singh P. K., Sharma S., Kumari A., Korpole S. (2014). A non-pediocin low molecular weight antimicrobial peptide produced by Pediococcus pentosaceus strain IE-3 shows increased activity under reducing environment. *BMC Microbiology*.

[16] Kumar B., Balgir P. P., Kaur B., Garg N. (2011). Cloning and expression of bacteriocins of Pediococcus spp.: A review. *Archives of Clinical Microbiology*.

[17] Gálvez A., Abriouel H., López R. L., Omar N. B. (2007). Bacteriocin-based strategies for food biopreservation. *International Journal of Food Microbiology*.

[18] Balciunas E. M., Castillo Martinez F. A., Todorov S. D., Franco B. D. G. D. M., Converti A., Oliveira R. P. D. S. (2013). Novel biotechnological applications of bacteriocins: A review. *Food Control*.

[19] Othman M., Ariff A. B., Wasoh H., Kapri M. R., Halim M. (2017). Strategies for improving production performance of probiotic Pediococcus acidilactici viable cell by overcoming lactic acid inhibition. *AMB Express*.

[20] Othman M., Ariff A. B., Rios-Solis L., Halim M. (2017). Extractive fermentation of lactic acid in lactic acid bacteria cultivation: A review. *Frontiers in Microbiology*.

[21] Ming L. C., Halim M., Rahim R. A., Wan H. Y., Ariff A. B. (2016). Strategies in fed-batch cultivation on the production performance of Lactobacillus salivarius I 24 viable cells. *Food Science and Biotechnology*.

[22] Abbasiliasi S., Tan J. S., Tengku Ibrahim T. A. (2017). Fermentation factors influencing the production of bacteriocins by lactic acid bacteria: A review. *RSC Advances*.

[23] Pattnaik P., Grover S., Batish V. K. (2005). Effect of environmental factors on production of lichenin, a chromosomally encoded bacteriocin-like compound produced by Bacillus licheniformis 26L-10/3RA. *Microbiological Research*.

[24] Kaboré D., Thorsen L., Nielsen D. S. (2012). Bacteriocin formation by dominant aerobic sporeformers isolated from traditional maari. *International Journal of Food Microbiology*.

[25] Benkeblia D. K., Lanzotti K. (2007). Application of bacteriocins in food preservation and safety. *Food*.

[26] Abbasiliasi S., Tan J. S., Ibrahim T. A. T. (2012). Isolation of Pediococcus acidilactici Kp10 with ability to secrete bacteriocin-like inhibitory substance from milk products for applications in food industry. *BMC Microbiology*.

[27] Abbasiliasi S., Tan J. S., Kadkhodaei S. (2016). Enhancement of BLIS production by Pediococcus acidilactici kp10 in optimized fermentation conditions using an artificial neural network. *RSC Advances*.

[28] Udhayashree N., Senbagam D., Senthilkumar B., Nithya K., Gurusamy R. (2012). Production of bacteriocin and their application in food products. *Asian Pacific Journal of Tropical Biomedicine*.

[29] Barcéna J. M. B., Sin F., de Llano D. G., Rodri A. N. A. (1998). Chemostat production of plantaricin C by *Lactobacillus plantarum* LL441. *Applied and Environmental Microbiology*.

[30] Tagg J. R., McGiven A. R. (1971). Assay system for bacteriocins. *Journal of Applied Microbiology*.

[31] Miao J., Guo H., Ou Y. (2014). Purification and characterization of bacteriocin F1, a novel bacteriocin produced by Lactobacillus paracasei subsp. tolerans FX-6 from Tibetan kefir, a traditional fermented milk from Tibet, China. *Food Control*.

[32] Yang E., Fan L., Yan J. (2018). Influence of culture media, pH and temperature on growth and bacteriocin production of bacteriocinogenic lactic acid bacteria. *AMB Express*.

[33] Todorov S. D., Rachman C., Fourrier A. (2011). Characterization of a bacteriocin produced by Lactobacillus sakei R1333 isolated from smoked salmon. *Anaerobe*.

[34] Partanen L., Marttinen N., Alatossava T. (2001). Fats and fatty acids as growth factors for Lactobacillus delbrueckii. *Systematic and Applied Microbiology*.

[35] Cálix-Lara T. F., Duong T., Taylor T. M. (2012). Addition of a surfactant to tryptic soy broth allows growth of a Lactic Acid Bacteria food antimicrobial, Escherichia coli O157:H7, and Salmonella enterica. *Letters in Applied Microbiology*.

[36] Settanni L., Valmorri S., Suzzi G., Corsetti A. (2008). The role of environmental factors and medium composition on bacteriocin-like inhibitory substances (BLIS) production by Enterococcus mundtii strains. *Food Microbiology*.

[37] Maria M., Janakiraman S. (2012). Detection of heat stable bacteriocin from Lactobacillus acidophilus NCIM5426 by liquid chromatography/mass spectrometry. *Indian Journal of Science and Technology*.

[38] Todorov S. D., Dicks L. M. T. (2006). Effect of medium components on bacteriocin production by Lactobacillus plantarum strains ST23LD and ST341LD, isolated from spoiled olive brine. *Microbiological Research*.

[39] Fahim H. A., Rouby W. M., El-Gendy A. O., Khairalla A. S., Naguib I. A., Farghali A. A. (2017). Enhancement of the productivity of the potent bacteriocin avicin A and improvement of its stability using nanotechnology approaches. *Scientific Reports*.

[40] Hwanhlem N., Chobert J.-M., H-Kittikun A. (2014). Bacteriocin-producing lactic acid bacteria isolated from mangrove forests in southern Thailand as potential bio-control agents in food: Isolation, screening and optimization. *Food Control*.

[41] Anthony T., Rajesh T., Kayalvizhi N., Gunasekaran P. (2009). Influence of medium components and fermentation conditions on the production of bacteriocin(s) by Bacillus licheniformis AnBa9. *Bioresource Technology*.

[42] Balciunas E. M., Al Arni S., Converti A., Leblanc J. G., Oliveira R. P. D. S. (2016). Production of bacteriocin-like inhibitory substances (BLIS) by Bifidobacterium lactis using whey as a substrate. *International Journal of Dairy Technology*.

[43] Renye J. A., Somkuti G. A., Garabal J. I., Steinberg D. H. (2016). Bacteriocin production by Streptococcus thermophilus in complex growth media. *Biotechnology Letters*.

[44] Yang R., Ray B. (1994). Factors influencing production of bacteriocins by lactic acid bacteria. *Food Microbiology*.

[45] Leroy F., Verluyten J., Messens W., De Vuyst L. (2002). Modelling contributes to the understanding of the different behaviour of bacteriocin-producing strains in a meat environment. *International Dairy Journal*.

[46] Liu W., Zhang L., Yi H. (2017). Development of a chemically defined medium for better yield and purification of enterocin Y31 from enterococcus faecium Y31. *Journal of Food Quality*.

[47] Strompfová V., Lauková A. (2007). In vitro study on bacteriocin production of Enterococci associated with chickens. *Anaerobe*.

[48] Callewaert R., De Vuyst L. (2000). Bacteriocin production with Lactobacillus amylovorus DCE 471 is improved and stabilized by fed-batch fermentation. *Applied and Environmental Microbiology*.

[49] Hartmann H. A., Wilke T., Erdmann R. (2011). Efficacy of bacteriocin-containing cell-free culture supernatants from lactic acid bacteria to control *Listeria monocytogenes* in food. *International Journal of Food Microbiology*.

[50] He L., Chen W., Liu Y. (2006). Production and partial characterization of bacteriocin-like pepitdes by Bacillus licheniformis ZJU12. *Microbiological Research*.

[51] Grazia S. E., Sumayyah S., Haiti F. S., Sahlan M., Heng N. C. K., Malik A. (2017). “Bacteriocin-like inhibitory substance (BLIS) activity of Streptococcus macedonicus MBF 10-2 and its synergistic action in combination with antibiotics. *Asian Pacific Journal of Tropical Medicine*.

[52] Anastasiadou S., Papagianni M., Filiousis G., Ambrosiadis I., Koidis P. (2008). Growth and metabolism of a meat isolated strain of Pediococcus pentosaceus in submerged fermentation. *Enzyme and Microbial Technology*.

[53] Aasen I. M., Møretrø T., Katla T, Axelsson L., Storrø I I. (2000). Influence of complex nutrients, temperature and pH on bacteriocin production by Lactobacillus sakei CCUG 42687. *Applied Microbiology and Biotechnology*.

[54] Albano H., Todorov S. D., van Reenen C. (2007). Characterization of two bacteriocins produced by Pediococcus acidilactici isolated from “Alheira”, a fermented sausage traditionally produced in Portugal. *International Journal of Food Microbiology*.

[55] Bali V., Panesar P. S., Bera M. B., Kennedy J. F. (2016). Bacteriocins: Recent Trends and Potential Applications. *Critical Reviews in Food Science and Nutrition*.

[56] Abdelsamei H. M., Ibrahim E. M. A., El Sohaimy S. A., Saad M. A. (2015). Effect of storage on the activity of the bacteriocin extracted from Lactobacillus acidophilus. *Benha Veterinary Medical Journal*.

[57] Ogunbanwo S. T., Sanni A. I., Onilude A. A. (2003). Characterization of bacteriocin produced by Lactobacillus plantarum F1 and Lactobacillus brevis OG1. *African Journal of Biotechnology*.

[58] Surovtsev V. I., Borzenkov V. M., Levchuk V. P. (2015). Purification of bacteriocins by chromatographic methods. *Applied Biochemistry and Microbiology*.

[59] Zhao R., Duan G., Yang T., Niu S., Wang Y. (2015). Purification, characterization and antibacteria mechanism of bacteriocin from Lactobacillus acidophilus XH1. *Tropical Journal of Pharmaceutical Research*.

[60] He Z., Tan J. S., Abbasiliasi S. (2015). Primary recovery of miraculin from miracle fruit, Synsepalum dulcificum by AOT reverse micellar system. *LWT- Food Science and Technology*.

[61] Yi L., Wu J. (2016). Purification and characterization of a novel bacteriocin produced by Lactobacillus crustorum MN047 isolated from koumiss from Xinjiang China. *Journal of Dairy Science*.

[62] Kaškonienė V., Stankevičius M., Bimbiraitė-Survilienė K. (2017). Current state of purification, isolation and analysis of bacteriocins produced by lactic acid bacteria. *Applied Microbiology and Biotechnology*.

